# Live attenuated influenza virus vaccine reduces virus shedding of newborn piglets in the presence of maternal antibody

**DOI:** 10.1111/irv.12531

**Published:** 2018-02-04

**Authors:** Marika Genzow, Christa Goodell, Troy J. Kaiser, Wesley Johnson, Marc Eichmeyer

**Affiliations:** ^1^ Boehringer Ingelheim Animal Health GmbH Ingelheim Germany; ^2^ Boehringer Ingelheim Animal Health St. Joseph MO USA; ^3^ Boehringer Ingelheim Animal Health Ames IO USA

**Keywords:** influenza A virus, live attenuated influenza virus vaccine, maternally derived antibodies, piglets, virus shedding

## Abstract

**Background:**

Influenza A virus in swine (IAV‐S) causes an acute respiratory disease of swine which results in great economic losses in pig production. Major control strategies include the use of killed vaccines (KV) in breeding females to confer passive immunity to their offspring. A bivalent H1N1 and H3N2 NS1‐truncated live attenuated IAV‐S vaccine have recently become available, which showed promising results in young pigs.

**Objective:**

The aim of this study was to investigate the effect of an intranasal vaccination of newborn pigs with or without maternally derived antibodies (MDA) on virus shedding (via nasal swabs tested by virus isolation).

**Methods:**

The study was performed as intratracheal challenge experiments with either a heterologous H1N2 or H3N2 viruses.

**Results and conclusion:**

The results of this study showed a significant decrease in the incidence and duration of shedding viable virus for vaccinated newborn piglets with or without MDA, providing strong evidence that intranasal vaccination is overcoming passively acquired maternal immunity. This study indicates that intranasal vaccination with a truncated NS1 live attenuated IAV‐S vaccine of newborn piglets with maternal antibodies can be a valuable tool for reducing the prevalence of heterologous H1N2 and H3N2 IAV‐S in pig herds.

## 1 INTRODUCTION

Influenza type A virus (IAV‐S) of the family Orthomyxoviridae causes an acute respiratory disease which results in distinct economic damages in global pork production. Control strategies include understanding of animal movements and IAV‐S circulation and subsequent establishment of appropriate biosecurity measures and vaccination strategies. The current standard of vaccination is using whole killed virus vaccines (KV), whereby KVs are most effective if the included strains closely match the currently circulating strains in pigs. KVs are used primarily in breeding females to confer passive immunity to their offspring, which does not protect piglets against infection and transmission of IAV‐S but rather against clinical disease).^1^ Recently, a live attenuated influenza virus vaccine (LAIV) has been reported to be efficacious in heterologous challenges in young piglets.^2^ Modifications introduced into the viral NS1 gene via reverse genetics have resulted in modified live influenza viruses that have been shown to be efficacious.^3^, ^4^ The aim of this study was to investigate whether vaccination with this LAIV would reduce viral shedding (duration and incidence) in newborn piglets with or without maternally derived antibodies (MDA).

## 2 MATERIALS AND METHODS

### 2.1 Vaccines and challenge virus

The IAV‐S LAIV was formulated by combining two viruses, the previously described I‐A triple‐reassortant internal gene (TRIG) cluster H3N2^5^ and a novel α‐cluster H1N1 which was constructed using the same techniques. Once combined, the viruses were lyophilized to create a bivalent vaccine. The vaccine was administered at a dose of 1 mL intranasally to one nostril.

IAV‐S challenge isolates γ‐cluster H1N2 (A/Swine/NorthCarolina/001169/2006) and cluster IV H3N2 (A/Swine/Nebraska/97901‐10/2008) were propagated in embryonated chicken eggs. Challenge doses were as follows: H1@4 = 2‐mL dose at 8.67 logs/mL, H3@5 = 2‐mL dose at 8.16 logs/mL, and H1@9 = 4‐mL dose at 8.49 logs/mL. The challenge material was kept on ice until administered per the EU Pharmacopeia: Pigs were manually restrained without anesthesia, while a catheter was passed just proximal to the tracheal bifurcation for administration of challenge material.

### 2.2 Experimental design

All study procedures and animal care activities were conducted at the American Association for Accreditation of Laboratory Animal Care (AAALAC)‐accredited Boehringer Ingelheim Sioux Center BL2 Facility in accordance with the ethical guidelines of the Boehringer Ingelheim Institutional Animal Care and Use Committee.

The primary objective was to determine the response to IAV challenge in pigs with MDA after vaccination with LAIV. Challenge time points were chosen to represent industry‐reported ages when commercial pigs were frequently diagnosed with IAV‐S in the field.^6^ Twenty (20) cross‐bred pregnant sows were sourced from a herd diagnosed with an IAV‐S clinical episode and subsequent vaccination of adult animals with a commercially available killed IAV product (FluSure, Zoetis Inc., Kalamazoo, MI, USA) within the previous 18 months (MidWest Farms, Burlington, CO, USA). Dams were tested for IAV nucleoprotein (NP) by MultiS‐Screen Ab ELISA (IDEXX Laboratories Inc., Westbrook, ME, USA) at Iowa State University Veterinary Diagnostic Laboratory; the NP ELISA assay is a blocking ELISA which reports results as S/N (sample to negative control) ratios using a cutoff of S/N <0.60 as positive. S/N results of dams range from 0.336 to 0.083. Sows were randomized to one of two rooms, and then, the treatment (control or vaccine) was randomized to room. As an additional challenge control source of piglets, seronegative sows were also obtained and housed separately from the seropositive sows (Wilson Prairie View Farms, Burlington, WI, USA). Ten dams tested negative for IAV NP by MultiS‐Screen Ab ELISA at ISU‐VDL and were randomized to one of two additional rooms, and all study activities occurred in parallel with the seropositive group. Sows farrowed between September 20, 2015, and September 25, 2015. Pigs were processed within 12 hours of birth which included a label dose of Excede for Swine (Zoetis, Lot #SF0233 exp 02/2017), Clostratox BCD (Novartis, either Lot #73‐1128B exp January 30, 2018, or Lot #73‐1129 exp March 18, 18), and iron supplementation (Uniferon 200, Lot #P860 exp 04/2017); processing did not include castration, needle‐teeth clipping, or tail docking. Between three and 7 days of age, all pigs were tested by NP ELISA: All pigs in the seropositive groups tested S/N ≤0.50 (with the exception of six pigs, four of which were littermates), and all pigs in the seronegative groups tested S/N ≥ 0.69. Pigs in the control groups then received a 1‐mL intranasal dose of saline as control, and pigs in the vaccine groups received a 1‐mL intranasal dose of bivalent vaccine rehydrated with diluent. Pigs were weaned 16 days after vaccination by removal of the sows from crates so that the pigs remained housed with littermates while awaiting randomization for the three respective challenge phase rooms.

To assess protection of the pigs in the presence of maternal antibodies over time, three different groups were evaluated post‐vaccination at 4, 5, and 9 weeks. For the first study time point, three pigs from each litter were randomly chosen for challenge with H1N2. At least 3 days prior to challenge, pigs were moved to a single room containing 10 identical pens; each pen contained a group of three littermates from the seropositive vaccine group commingled with a group of three littermates from the seropositive control group. At 4 weeks post‐vaccination, all pigs in the first group were challenged with H1N2 (H1@4). From the remaining pigs, three pigs from each litter were randomly chosen for the second challenge group with H3N2 at 5 weeks post‐vaccination (H3@5) and commingled by the same manner. The remaining pigs made up the cohort for the third challenge group with H1N2 at 9 weeks post‐vaccination (H1@9). Three vaccinated pigs and four or five seropositive control pigs were commingled into a single pen (as litter sizes were uneven, some litters were split between pens). For each study time point, the same design (in a separate room) was applied to seronegative pigs with two pigs per litter randomized to the H1@4 and H3@5 challenge studies and the remainder included in H1@9 challenge study (Table 1).

**Table 1:** Experimental design

Serostatus of dams (MDA)	Number of piglets	Treatment	Challenge strain	DPV (wk)	Group name
Seropositive	30	Control	H1N2	Day 31 (week 4)	H1@4
Seropositive	30	Vaccinate			
Seronegative	14	Control			
Seronegative	12	Vaccinate			
Seropositive	30	Control	H3N2	Day 39 (week 5)	H3@5
Seropositive	30	Vaccinate			
Seronegative	14	Control			
Seronegative	12	Vaccinate			
Seropositive	53	Control	H1N2	Day 66 (week 9)	H1@9
Seropositive	30	Vaccinate			
Seronegative	14	Control			
Seronegative	13	Vaccinate			

### 2.3 Observations and sampling

Pigs were monitored daily for general demeanor, respiratory signs, and rectal temperature from −2 to 5 days post‐challenge (dpc). Nasal swabs were collected daily from −1 to 5 dpc by insertion of a single swab (Fisherbrand, poly tip Catalog No. 23‐400‐122) into each nostril. The swabs were then stored frozen at −70 °C in a 5‐mL tube containing 2 mL of tissue culture media formulated with antibiotic and antimycotic until testing could be completed.

### 2.4 Virus isolation

Twenty‐four well tissue culture plates of KEW‐MDCK cells (a MDCK cell line stably expressing the Influenza A NS1 gene, which has been useful for detecting low levels of IAV‐S in clinical samples) were seeded with 1 mL of growth media, minimum essential media (MEM) modified (SAFC Cat # 62892‐1000M3056) with 5% fetal bovine serum (SAFC Cat # 12003C‐1000ML) containing 1 x 10^5^ cells. The plates were then incubated for 4 days at 37°C with 4.5% carbon dioxide. After incubation, the plates were prepared for testing by decanting the growth media and adding back 0.5 mL of wash media (MEM modified without serum but instead containing 2 units/mL of porcine trypsin (SAFC Cat # T5266‐500 mg)). The plates were then incubated at 37°C with 4.5% carbon dioxide, while the samples were being thawed at room temperature. Next, 0.50‐0.75 mL of each sample was transferred to a centrifuge tube and spun at 10 000x *g* for 2 minutes in order to pellet sample debris. The wash media were then decanted from the KEW‐MDCK plates, and approximately 100 μL of centrifuged sample was dispensed through a 0.2‐μm filter (Pall Cat # 4602) into each of two duplicate wells. The plates were then incubated at 37°C with 4.5% carbon dioxide for 1 hour; after this incubation, 0.5 mL of wash media was added back to each well. The plates were then incubated at 37°C with 4.5% carbon dioxide for 7 days to allow for low levels of virus to replicate and amplify to levels that can be detected in a hemagglutination assay. After incubation, the supernatant of the duplicate sample wells was harvested and pooled. Then, 50 μL of the pooled material was dispensed into duplicate wells of a 96‐well round bottom plate (BD Falcon Cat # 353910). Washed male turkey red blood cells (Lampire Biological Laboratories Cat # 7209603) diluted to a 0.5% concentration were added to each sample well at 50 μL/well. The plates were sealed and incubated at room temperature for approximately 1 hour or until the negative control wells formed a button and the positive control wells formed a mat.

### 2.5 Statistics

The null hypothesis was that vaccination would not result in difference with regard to the presence of viable virus in nasal swab results for vaccinated pigs compared to non‐vaccinated control pigs. Results were evaluated by means of a repeated‐measures two‐way ANOVA with SAS version 9.4 (Cary, NC, USA). Measurements of frequencies were evaluated by means of the chi‐square test. Results were considered significant if *P* ≤ .05.

## 3 RESULTS

### 3.1 NP ELISA

Prior to treatment, all pigs were tested to confirm their serostatus (positive or negative) by the NP ELISA (Table 2). The serostatus was investigated again prior to either challenge. There was a decline in titers in all seropositive groups as evidence of a decay of maternal antibodies. The results also show that following intranasal vaccination, there was little to no seroconversion in all three experiments.

**Table 2:** NP ELISA S/N titers at treatment and before challenge

Serostatus of dams (MDA)	Number of piglets	Treatment	Challenge strain	DPV (wk)	Group name	Median NP ELISA S/N at treatment^a^	Median NP ELISA S/N titer at challenge^a^
Seropositive	30	Control	H1N2	Day 31 (week 4)	H1@4	0.17	0.54
Seropositive	30	Vaccinate				0.13	0.51
Seronegative	14	Control				0.82	0.92
Seronegative	12	Vaccinate				0.86	0.87
Seropositive	30	Control	H3N2	Day 39 (week 5)	H3@5	0.17	0.62
Seropositive	30	Vaccinate				0.12	0.60
Seronegative	14	Control				0.83	0.90
Seronegative	12	Vaccinate				1.02	0.78
Seropositive	53	Control	H1N2	Day 66 (week 9)	H1@9	0.16	0.79
Seropositive	30	Vaccinate				0.12	0.81
Seronegative	14	Control				0.81	1.00
Seronegative	13	Vaccinate				0.94	0.90

a NP ELISA S/N result <0.60 = seropositive.

DPV, days post‐vaccination at which challenge was administered.

### 3.2 Respiratory signs and body temperature

Pigs showed a notable increase in respiratory effort 2 days after challenge in all groups; however, the control groups had more pigs with severe dyspnea than the vaccinated groups. Respiratory signs lasted for not longer than 4 dpc in all groups (data not shown).

**Figure 1:**
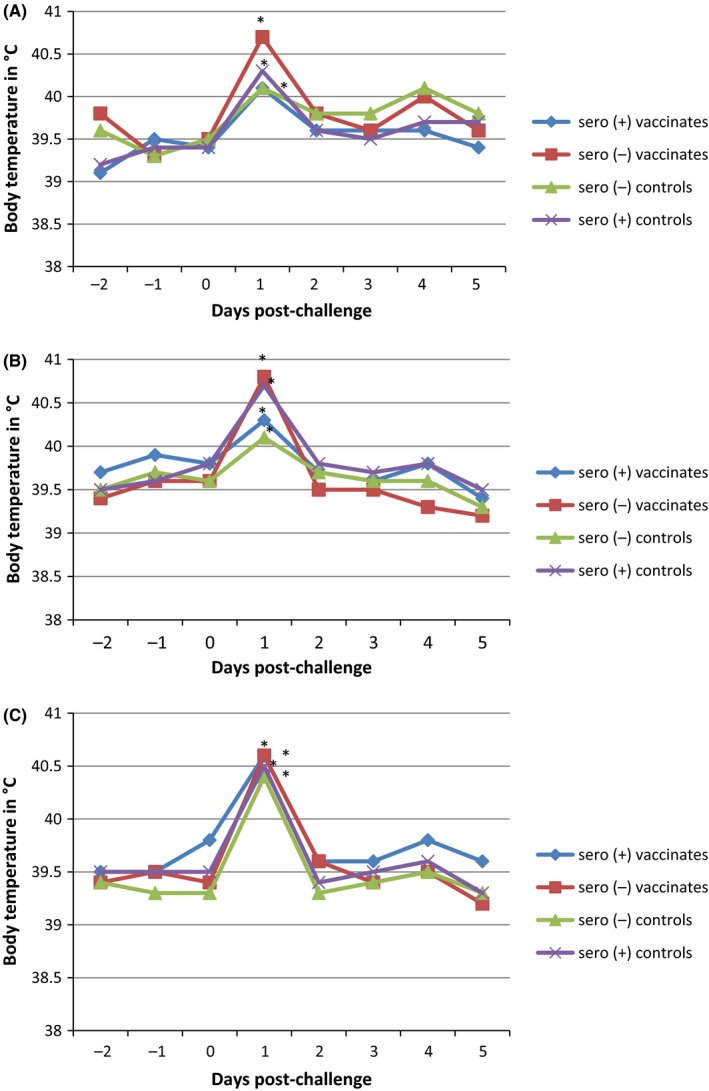
LSMean course of body temperature by treatment group for the three challenge events (*P ≤ .05 ANOVA). (A) First H1N2 challenge (4 wk of age). (B) H3N2 Challenge (5 wk of age). (C) Second H1N2 challenge (9 wk of age)

All three challenge events (H1@4, H3@5, and H1@9) resulted in a significant increase in body temperature compared to baseline for all treatment groups 1‐day post‐challenge (*P* ≤ .05) for both seropositive and seronegative animals (Figure 1A,B,C), lasting 1 day. This increase was correlated with an increase in respiratory signs. No significant elevation in body temperature in any group was observed after 4 dpc. At 5 dpc, the body temperature of all animals in all groups returned to baseline values.

### 3.3 Virus isolation of nasal swabs

**Figure 2:**
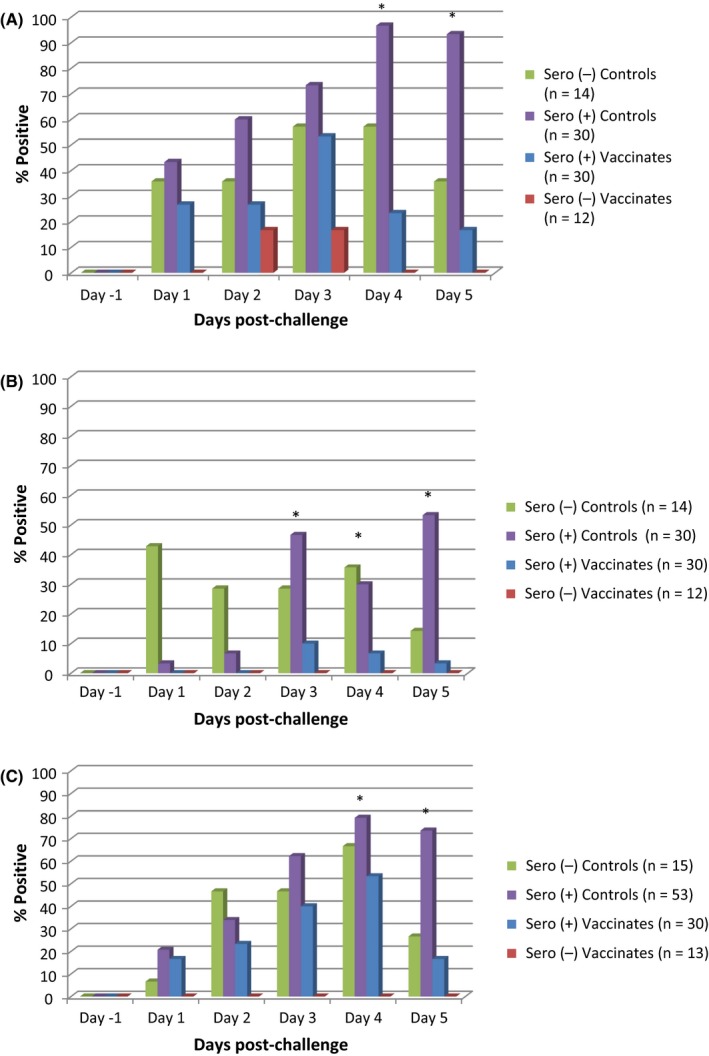
Number of virus‐positive nasal swabs by treatment group and day post‐challenge (*P ≤ .05 chi‐square test). (A) First H1N2 challenge (4 wk of age). (B) H3N2 challenge (5 wk of age). (C) Second H1N2 challenge (9 wk of age)

All pre‐challenge nasal swabs of all groups tested negative for IAV‐S. Animals in the control group had more virus‐positive nasal swabs compared to vaccinated groups, which was significant on 4 and 5 dpc in H1@4w (Figure 2A) and H1@9w (Figure 2C) and significant on 3, 4, and 5 dpc in H3@5w (*P* ≤ .05) (Figure 2B).

### 3.4 Median duration shedding over time

**Figure 3:**
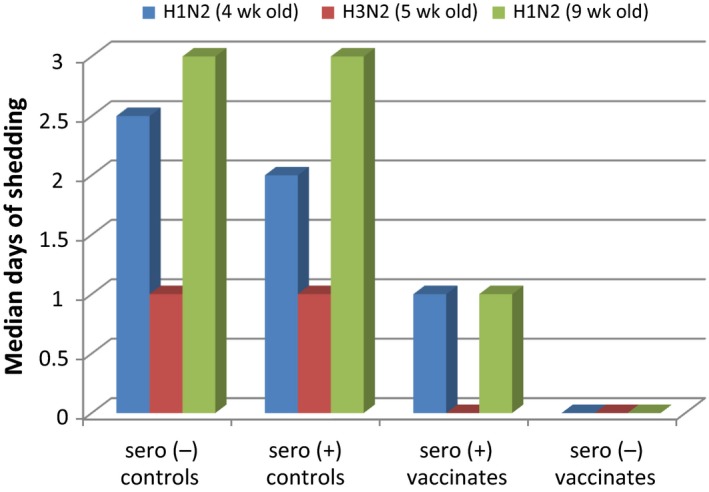
Median duration of virus shedding in nasal swabs

As evident from Figure 3, both vaccinated groups had the shortest duration of nasal virus shedding (as measured by virus isolation), compared to the seronegative and positive non‐vaccinated groups.

Seropositive vaccinated pigs had a median duration of shedding of 1 day in both H1@4 and H1@9 and no shedding in H3@5. The median duration of shedding was lower in the H3N2 challenge compared to both H1N2 challenges.

## 4 DISCUSSION

Modifications introduced into the viral NS1 gene via reverse genetics have resulted in an LAIV with promising immunological and laboratory results.^3^, ^7^, ^8^ While it is known that KVs used in breeding females confer only passive immunity to their offspring, understanding the effects of an intranasally applied LAIV to newborn piglets with regard to virus shedding (as measured by virus isolation) was crucial to further investigate the effect on possible virus transmission. As most studies requested from regulatory bodies must be conducted in MDA‐negative pigs, it was of great interest to investigate the shedding pattern when vaccinated MDA‐positive newborn piglets are exposed to virulent IAV‐S strains.

The three independent challenge studies (H1@4, H3@5 and H1@9) were performed successfully as evidenced by increase in body temperature and increase in respiratory signs following intratracheal administration of either IAV‐S virus in all treatment groups. The intranasal administration of the LAIV resulted in little to no seroconversion, which can be explained that intranasal vaccination with an attenuated virus is considered likely to elicit more cross‐reactive T cells and mucosal antibodies against antigenically variant strains than those induced by other types of vaccination.^9^ Our findings are in agreement with previous studies.^2^

As shown from the virus isolation results, vaccination of MDA‐positive piglets resulted in a significant reduction in the incidence and duration of viable virus in nasal secretions. This was true for all three challenge groups. The significantly elevated number of animals shedding after 2dpc in MDA‐positive non‐vaccinated pigs of all three challenge groups was notable, possibly suggestive of suboptimal protection and the absence of cross‐neutralizing antibodies, factors associated with vaccine‐associated enhanced respiratory disease (VAERD);^10^ however, pathological evidence of VAERD was not identified in any treatment group. Possibly related to this observation, over 90% of the pigs in the MDA seropositive unvaccinated pigs were shedding virus at 4 and 5dpc, while that percent was 70% in the H1@9 challenge group, when MDA in those older pigs had declined.

While the reduction in virus shedding as shown by virus isolation was greatest in the MDA‐negative vaccinated pigs, vaccination of MDA‐positive animals resulted in a distinct reduction in shedding of viable virus in seropositive pigs, demonstrating intranasal vaccination with this LAIV overcame maternal immunity.

Viral titer of each individual positive swab was not conducted; however, the authors suspect that as fewer vaccinated pigs shed IAV‐S for shorter duration, virus from nasal secretions from the vaccinated pigs was of lower IAV‐S concentration than those of control pigs, where more pigs shed viable virus for longer duration. No comparisons were made between challenge studies as two different isolates were used in this study and pathogenicity of each virus was not established prior to study initiation.

A recent study has shown that piglets play a key role in maintaining IAV‐S in the breeding herds,^11^ which provides evidence that early piglet vaccination with a vaccine that provides heterologous protection will contribute to reduce IAV‐S prevalence both in a herd and between herds. More studies are needed to quantify the reduction in prevalence by including more systems of different sizes and management practices.

In conclusion, this study provides evidence that intranasal vaccination of MDA‐positive piglets with a truncated NS1 live attenuated influenza A vaccine resulted in significantly reduced viral replication and shedding after both H1N2 and H3N2 challenges.

## CONFLICT OF INTEREST

All authors are employees of Boehringer Ingelheim.
